# Epidemiological Study Related to the First Outbreak of Ovine Anaplasmosis in Spain

**DOI:** 10.3390/ani11072036

**Published:** 2021-07-08

**Authors:** Delia Lacasta, Miguel Lorenzo, José María González, Marta Ruiz de Arcaute, Alfredo Ángel Benito, Cristina Baselga, María Eugenia Milian, Nuria Lorenzo, Calasanz Jiménez, Sergio Villanueva-Saz, Luis Miguel Ferrer

**Affiliations:** 1 Animal Pathology Department, Instituto Agroalimentario de Aragón-IA2, Universidad de Zaragoza-CITA, 50013 Zaragoza, Spain; miguel_lof@hotmail.com (M.L.); jmgsovino@gmail.com (J.M.G.); martarda@unizar.es (M.R.d.A.); svs@unizar.es (S.V.-S.); lmferrer@unizar.es (L.M.F.); 2 Centro Clínico Veterinario de Jaca, C/Estudios 5, 22700 Jaca, Spain; 3 Exopol S.L., Pol. Río Gállego D-8, 50840 Zaragoza, Spain; abenito@exopol.com (A.Á.B.); crbaselga@exopol.com (C.B.); 4 A.D.S. Bajo Aragón Matarraña, 44596 Teruel, Spain; memilian@oviaragon.com; 5 A.D.S. ovina y caprina del Matarraña Turolense, 44596 Teruel, Spain; nuria.lorenzo.a@gmail.com; 6 Gabinete Técnico Veterinario S.L., C/Isla conejera sn, 50013 Zaragoza, Spain; calasanz.jimenez@gmail.com

**Keywords:** ovine anaplasmosis, *Anaplasma ovis*, epidemiological study, emerging disease

## Abstract

**Simple Summary:**

Ovine anaplasmosis is an emerging disease in Europe, able to cause relevant economic losses. This disease has great significance in tropical and subtropical areas; however, climate change has facilitated the wider spread of ovine anaplasmosis throughout Europe during the last few decades. Related to the first ovine anaplasmosis outbreak that occurred in Spain in 2014, an epidemiological study was carried out in the affected area. The results show a high presence of *Anaplasma ovis* in the analysed flocks, indicating the high transmissibility of the bacteria.

**Abstract:**

Ovine anaplasmosis is a vector-borne disease caused by *Anaplasma ovis* and mainly transmitted through tick bites. In Spain, the first outbreak of ovine anaplasmosis occurred in 2014. An epidemiological study in fifty-one farms was carried out associated with this outbreak in the affected geographical area. An epidemiological questionnaire was performed. In addition, whole blood samples were taken for molecular analysis in 47 of these farms to determine the prevalence of infection of *Anaplasma ovis*. *A. ovis* was present in 44 out of 47 PCR-analysed farms (93.6%). However, only 40.4% of the studied farms showed severe clinical signs. The clinical signs affected mainly young animals, which showed severe anaemia, weakness, anorexia, cachexia and epiphora. The early culling of young animals was more frequently reported by severely affected farms than the analysed farms without clinical signs (71.4% vs. 12.5%, *p* < 0.001). The geographical area where the farm is located seems to be relevant for the presence of clinical signs of the disease. Ovine anaplasmosis is an emerging disease in Europe that spreads rapidly through tick bites and is capable of causing significant economic losses when it spreads in a naive area and causes an epidemic.

## 1. Introduction

Ovine anaplasmosis is an emerging disease in Europe, caused by the bacteria *Anaplasma ovis*, that is transmitted through tick bites. This disease has great significance in developing countries in tropical and subtropical areas. During the last few decades, climate change has facilitated the wider dissemination of ovine anaplasmosis, which is being spread very quickly throughout Europe. In recent years, it has been reported in many different European countries, such as Bulgaria, France, Germany, Greece, Hungary, Italy, Portugal, Romania and Turkey [1,2,3]. In Spain, *A. ovis* infection was reported for the first time in roe deer (*Capreolus capreolus*) [4]. The first disease outbreak in sheep associated with severe clinical signs due to *A. ovis* has only been recently described by our research team [5,6]. The acute phase of the disease is characterised by nonspecific clinical signs such as weakness, depression, a marked loss of body condition, fever and progressive anaemia [5,7]. In lambs, the disease has been observed in the abattoir, with jaundiced carcasses that are condemned from entering the human food chain [6]. Therefore, the impacts of *A. ovis* are not only on animal welfare, rural livelihoods and farm sustainability but might also result in significant wastage of food intended for human consumption.

Bacteria of the *Anaplasma* genus belong to the order of the Rickettsiales and are obligate intracellular agents, causing vector-borne diseases in mammals. *A. ovis*, *A. marginale* and *A. centrale* infect erythrocytes in ruminants. *A. ovis* is a specific pathogen of sheep, although it can also be found in other species. *A. phagocytophilum* affects a large number of mammalian species, including sheep, causing human granulocytic anaplasmosis [8]. Anaplasmosis is currently considered an emerging zoonosis, and the number of cases in humans has recently increased significantly, mainly associated with *A. phagocytophilum*. There is a strong suspicion that anaplasmosis caused by *A. ovis* could also be a zoonosis [9].

*Anaplasma* genus bacteria are transmitted by mechanical and biological vectors, mainly ticks. Some studies have shown that up to 19 species of ticks can transmit the disease [10], depending on geographical location and seasonality. In Spain, the presence of *Anaplasma* bacteria in ticks of the *Rhipicephalus*, *Ixodes*, *Hyaloma*, *Dermacentor* and *Amblyomma* genera has been demonstrated [11]. In the salivary glands of ticks, bacteria replicate, increasing their infective capacity [12]. In other *Anaplasma* species, infectivity is achieved when there are 10^6^ microorganisms in ticks’ salivary glands [13]. This dose-dependent biological transmission has greater efficacy and, therefore, greater relevance than the mechanic. Within the erythrocyte, the bacterium replicates by binary fission to form up to eight individual organisms within a simple vacuole [14]. Anaplasma leaves the erythrocyte using a mechanism that has not been well defined, apparently non-lytic, to infect new erythrocytes. The destruction of erythrocytes and the consequent anaemia are not, however, immediate, with noticeable changes around 30–40 days post-infection [5]. The severe haemolytic anaemia associated with this disease is a consequence of the immune response caused by anaplasmosis infection [15].

The diagnostic technique used before the development of molecular techniques was the examination of whole blood in the form of a smear, in which vacuoles were observed in an optical microscope inside the erythrocytes [16]. However, this technique is now obsolete, and, currently, the most common diagnostic method is the polymerase chain reaction (PCR), since it is capable of detecting infections with a minimum of 0.0001% of infected erythrocytes. In addition, PCR-amplified products can be sequenced to differentiate the species of *Anaplasma*. Currently, the *msp4* gene, encoding a major membrane protein, is used to differentiate the different *Anaplasma* species [17]. Serological tests are limited to competition Enzyme-Linked Immunosorbent Assay cELISA methods that are designed to detect the MSP5 protein, which is present in all *Anaplasma* species. Currently, there is only one commercial kit for *A. marginale* that has demonstrated successful detection of *A. ovis* infection in sheep [18]. 

*Anaplasma ovis* is silently transmitted, not always causing obvious clinical signs. Furthermore, if left untreated, infected sheep remain carriers for life, favouring the spread of the infection. The present study was carried out in the geographic region where the first anaplasmosis outbreak occurred in Spain, at the time of occurrence. The main objective was to determine the collective prevalence of the infection in the region as well as to detect possible farm management factors that could have influenced the appearance of clinical signs. 

## 2. Materials and Methods

A severe outbreak of ovine anaplasmosis affecting a wide geographical area occurred for the first time in Spain in the Matarraña region (Aragón, Spain). Young animals around one year of age were the most severely affected, and the main clinical signs were severe anaemia, anorexia, depression and chronic weight loss. To determine the presence of *Anaplasma ovis* in the flocks of the area, samples were taken according to the size of the flocks and pools of 5 blood samples were made to detect the presence of *A. ovis* by means of qPCR.

The care and use of animals were performed in accordance with the Spanish Policy for Animal Protection RD53/2013, which meets the European Union Directive 2010/63 on the protection of animals used for experimental and other scientific purposes.

### 2.1. Epidemiological Questionnaire and Selected Farms

Fifty-one sheep farms in the Matarraña region voluntarily participated in the epidemiological survey performed from March to November 2015. An epidemiological questionnaire was distributed to each farmer, asking questions related to breed, flock size, reproductive management, grazing areas, feeding, health plans, diseases present in the flock, observation of anaplasmosis compatible symptoms, presence of vectors, etc. In addition, blood samples with anticoagulant (EDTA) were collected from the jugular vein through a vacutainer system and frozen at minus 20 °C before being sent to the laboratory for molecular analysis. 

**Figure 1:**
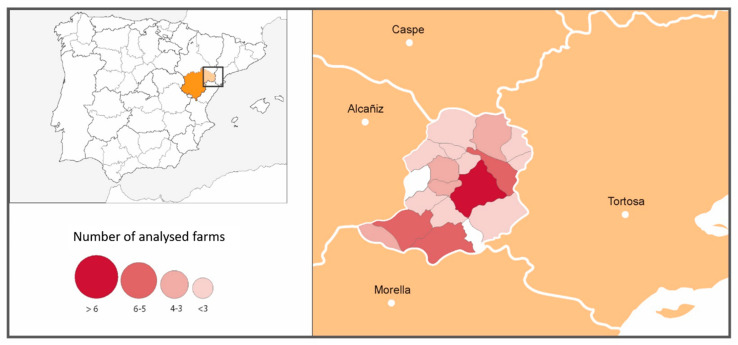
Number of farms sampled by municipalities within the Matarraña region.

A classification of the farms in three levels was performed according to the epidemiological survey, focused on the presence/absence of animals with compatible clinical signs and the use of antibiotics to treat them. Farms of type I were those without anaplasmosis-compatible clinical signs. Farms of type II had animals with compatible clinical signs, but no antibiotics had been administered. Finally, farms of type III had animals with compatible clinical signs that had been treated with antibiotics. In Figure 1, the number of farms sampled by municipalities within the Matarraña region is shown.

**Figure 2:**
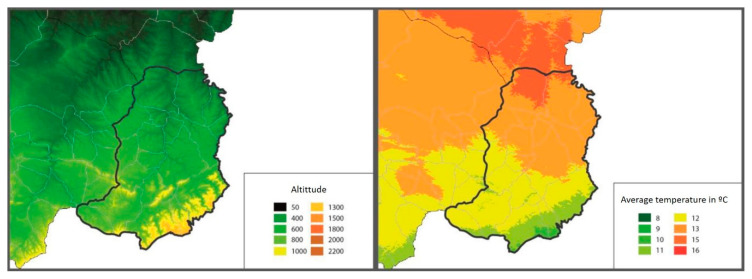
Maps of the Matarraña region, where the altitude (**left**) and average temperature in °C (**right**) are shown (http://www.opengis.uab.es/wms/aragon/. Access 15 May 2021).

In the Matarraña region, despite its small size (926.06 km^2^), there are two well-differentiated natural areas. The northern region, Bajo Matarraña, is characterised by a continental climate with open spaces with small elevations. The main crops are olive trees (Olea Europea), almond trees (Prunus dulcis) and rainfed cereal, interspersed with forests of Aleppo pine (Pinus halepensis). Meanwhile, the southern region, Alto Matarraña, has a higher altitude (700–1396 m), and the climate is more humid, with extensive forest formations of black pine (Pinus nigra) and Scots pine (Pinus sylvestris) [19] (Figure 2). 

### 2.2. Sample Size

From each flock, a sample pooling including five animals younger than five years, who were randomly selected, was performed as a strategy to detect the presence of *A. ovis* in each farm. Thus, in type I farms, it was decided to sample 30 animals analysed in 6 pools of five sheep. A prevalence lower than 10% was assumed to indicate a flock free from infection, with a 95% confidence interval. In type II farms, five animals analysed in one pool were sampled. In these farms, a very high prevalence of *A. ovis* was expected; then, those animals with compatible clinical signs were sampled. In this type of farm, previous studies have revealed that the expected prevalence, detected by PCR of “pools” of 5 samples, was greater than 90%. Again, a 95% confidence interval was assumed. Finally, in type III farms, 15 animals were sampled in three pools of five animals. Unlike the previous case, the prevalence detected in previous studies in this type of farm was lower due to the antibiotic action. As there was a high probability that the disease was present on the farm, it was decided to consider a flock free of *A. ovis* with a prevalence lower than 20%. Assuming a 95% confidence interval, the target population for sampling, in this case, included all animals younger than five years since the treatment received might have masked the clinical signs, preventing the selection of sick animals.

### 2.3. Haematological Analysis

Samples of blood with anticoagulant (EDTA) were collected from the jugular vein through a vacutainer system from fifty-two clinically affected animals randomly selected from affected farms. These animals were selected as they presented symptoms such as anorexia or pale mucous membranes. Haematology was performed using an automatic haematological counter Vet-ABC (DIVASA-FARMAVIC S.A., Barcelona, Spain). Measured parameters were leukocytes, erythrocytes, haemoglobin, haematocrit, platelets, mean corpuscular volume (MCV), mean corpuscular haemoglobin (MCH) and mean corpuscular haemoglobin concentration (MCHC). In addition, a microscopic evaluation of blood smears stained with 10% Giemsa was performed for the detection of *Anaplasma* organisms infecting erythrocytes.

### 2.4. PCR Analysis

The commercial kit, MagMAX™ Pathogen RNA/DNA (Thermo Fisher Scientific, Waltham, MA, USA), with an automated magnetic particle processor (KingFisher Flex System, Thermo Fisher Scientific), was used for nucleic acid extraction according to the manufacturer’s instructions. Amplification was carried out in a 7500 fast Real-Time PCR machine (Applied Biosystems, Waltham, MA, USA), and results were analysed with the respective software (7500 software v2.3).

A total of 864 blood samples were taken from 45 sheep farms (Table 1), although finally, only 111 pools were analysed by means of qPCR because as soon as a pool tested positive in one of the studied farms, analysis of the remaining samples from that flock was stopped. In six of the analysed flocks, blood samples could not be taken due to logistical reasons.

The specific detection of *Anaplasma ovis* was carried out by using the commercial kit EXOone *Anaplasma ovis* (EXOPOL S.L.) and following the manufacturer’s instructions. This qPCR assay has an analytical sensitivity of 50 copies of genomic equivalent/reaction and includes a quantified synthetic positive control. The assay targets the single copy MSP4 gene that is reported to allow a specific differentiation of *Anaplasma ovis* from the highly related *Anaplasma marginale* [20]. Additionally, an endogenous control was also included in all of the assays in order to avoid false-negative results. The bacterial load was expressed using the quantification cycle (Cq), which is the cycle number where the PCR amplification curve intersects the threshold line [21]. The Cq value can be used to quantify or to determine the presence/absence of the target sequence.

### 2.5. Statistical Analysis

All the information obtained in the epidemiological survey and in the different analytical tests was integrated into the same statistical matrix and processed with the statistical package SPSS 20.0 (SPSS Inc., Chicago, IL, USA) in order to determine the statistical relationship between the presence of *A. ovis* and clinical signs, as well as its possible risk factors. The farm was used as a study unit. The disease study was carried out by categorising the flocks according to clinical signs into three types: flocks without clinical signs of anaplasmosis, flocks with mild signs (less than 1% of young animals affected) and flocks with severe signs (more than 5% of infected young animals). In addition, flocks were classified according to the presence of *A. ovis* in the PCR-analysed samples as free or infected flocks. The study of the variables included in the survey, and the criteria for determining infection, were also analysed using non-parametric Chi-square tests. For those cases in which it was possible (2 × 2 tables), the relative risk was calculated. The values corresponding to numerical variables, such as counts obtained from haematologies, censuses, percentages of deviation, etc., were also analysed by ANOVA tests when the normality tests were passed or by non-parametric tests in cases that lacked normal distribution. The non-parametric tests used were Mann–Whitney for the presence of the etiological agent and Kruskall–Wallis for clinical criteria. Likewise, a logistic regression was carried out with the analysed data; however, due to the low number of sampled farms, no significant results were obtained. In all cases, results with *p* < 0.05 were assumed to be significant.

## 3. Results 

### 3.1. Epidemiological Survey

Twenty-one of the 51 farms surveyed during this study reported currently or previously having animals with clinical signs of severe anaplasmosis (21/51: 41.2%). One hundred per cent of the affected farmers had or previously had animals with weakness, anorexia and cachexia, and 95.2% (20/21) of these farmers reported having seen animals with epiphora among those affected. Other symptoms such as lameness, haematuria/haemoglobinuria or abortions were seen in a much lower proportion. In Table 2, it can be seen that weakness, anorexia and cachexia were clinical signs clearly associated with mild or severe anaplasmosis. However, epiphora and haematuria were only present in severe anaplasmosis cases, haematuria being observed only in 4.8% of animals affected by severe anaplasmosis. Lameness does not seem to be a symptom of anaplasmosis, and the number of abortions increased with the severity of the disease.

In addition, 71.4% (15/21) of the severely affected farms had a higher incidence of culling young (<3 years old) animals than farms without clinical signs (3/24, 12.5%; *p* < 0.001), but these levels were similar to farms that showed mild clinical signs of anaplasmosis (3/6; 50.0%; *p* = 0.367). The flocks with severe anaplasmosis had a 16.7-times (3.58–77.68) greater risk of culling young animals than those with no clinical signs of anaplasmosis.

In the studied area, three different sheep breeds were reared, Rasa Aragonesa, Ojinegra de Teruel and Maellana, all of them local breeds that are very well adapted to the type of grazing and climate of the area. The study results do not show significant differences in the presence of anaplasmosis clinical signs between the different breeds. In Table 3, the presence of clinical anaplasmosis is analysed in relation to the breed. 

**Figure 3:**
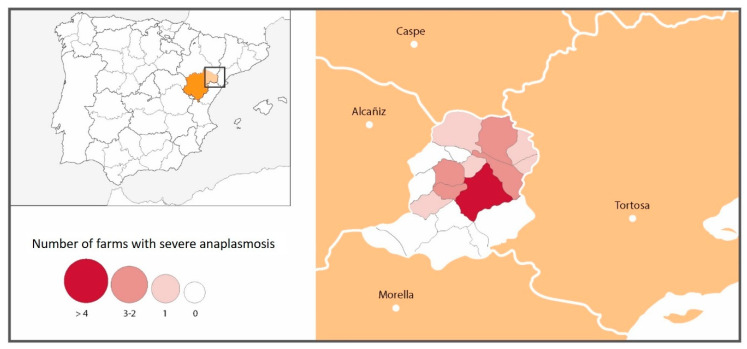
Number and location of farms with severe anaplasmosis.

Although clinical signs of anaplasmosis were observed throughout the whole Matarraña region, severe clinical signs were seen much more frequently in farms located in the north of the province (21/36 north vs. 0/15 south *p* < 0.001) (Figure 3, Table 4). The risk of having severe anaplasmosis in the north was 2.0-times (2.86–1.40) higher than in the south of the region.

Likewise, the average altitude at which those farms that suffered severe anaplasmosis were located was 128 m lower than those showing the absence of symptoms (652 m in flocks without anaplasmosis vs. 623 m in mild anaplasmosis and 524 m in those flocks with severe anaplasmosis) (*p* = 0.056).

The presence of ticks in the entire area was very relevant. During the visits to the farms, sporadic samples of ticks found on the animals were collected, and in all cases, the identified species were Rhipicephalus turanicus (Dr. Estrada-Peña’s identification). Ninety-two per cent of the farmers surveyed (46/50) saw ticks on their animals, and 78.0% (39/50) observed horseflies, spring being the time at which the highest numbers were observed. Furthermore, 36.0% (18/50) said that the number of ticks had increased in recent years. The relationship between seeing an increase in vectors and the presentation of severe anaplasmosis was significant (11/18 vs. 10/32, *p* = 0.040). Finally, the risk was 3.5-times (1.03–11.56) higher in the group of farms that reported an increase in the number of ticks. Table 5 shows the increased presence of ticks in the last few years reported by the farmers and the absence of treatment for ticks in those farms without anaplasmosis or with mild or severe clinical signs of the disease. 

### 3.2. Haematological Analysis

Forty-six out of 52 analysed sheep with clear anaplasmosis clinical signs (88.5%) showed severe normochromic normocytic anaemia. In addition, six animals showed leukopenia (11.5%) and only one (1.9%) presented leukocytosis (Table 6). 

### 3.3. Anaplasma ovis Detection by qPCR

Forty-five out of the 51 farms visited were finally analysed using molecular techniques (qPCR). Forty-one of the 45 analysed farms showed at least one blood pool positive for *Anaplasma ovis* (91.1%); in 36 of these farms, all the pools tested positive (87.8%). In addition, twenty-one (87.5%) out of twenty-four flocks that showed clinical signs had positive PCR results, as well as 19 of the 20 without clinical signs (95.0%) (Table 7). 

## 4. Discussion

Ovine anaplasmosis caused by *Anaplasma ovis* is a disease transmitted by vectors, mainly ticks, that is widespread in tropical and subtropical areas. This disease is frequently diagnosed in countries such as Brazil, Mexico, Iran and India, with a prevalence more significant than 50%, followed by others such as Morocco and Tunisia, with a prevalence between 20% and 50%, or China, with results ranging between 9.4% and 65.3% [22,23]. However, in recent years, the number of references for this disease in Europe has increased exponentially; the disease has been described in different European countries [1,3,24,25], indicating the rapid spread of *A. ovis* throughout the continent. In Spain, *A. ovis* infection was detected in roe deer in 2008 [4] and sheep in 2013 [26]. However, the first disease outbreak was recently described by our research team [5,6]. Epidemiological studies assessing the distribution of *A. ovis* across Europe are lacking, and no epidemiological studies have been developed in Spain so far. In the present study, *A. ovis* was present in 41 out of 45 PCR-analysed farms, which means a collective prevalence in the small ruminant farms of the Matarraña region of 91.1%. However, three of the flocks with clinical signs of anaplasmosis were PCR-negative. This could be due to the fact that farmers experiencing a severe outbreak of the disease treated the animals with antibiotics, mainly oxytetracycline and doxycycline. Although it has not yet been reported in the ovine literature, based on the authors’ experience extrapolated from the use of these antibiotics in other species, treatment with antibiotics, especially doxycycline, destroys the bacteria, causing sick animals to become *A. ovis* PCR-negative.

The main biological vectors of anaplasmosis are ticks, mainly of the genera *Rhipicephalus (R. bursa* and *R. turanicus)*, *Dermacentor (D. silvarum*, *D. marginatus* and *D. andersoni)*, *Ixodes* and *Ambliyomma* [10,11,27,28,29]. In our study, the only tick species found in all the samples taken was *Rhipicephalus turanicus*. Moreover, 92.0% of the surveyed farmers reported detecting the presence of ticks on their animals, especially in spring, and 36.0% said that the number of ticks had increased in recent years. It was observed that the relationship between seeing an increase in vectors and the presentation of severe anaplasmosis in the farm was significant (*p* < 0.05), with a 3.77-times more risk of suffering anaplasmosis among farmers who detected an increase in the number of ticks. However, this assessment could have a certain degree of subjectivity since most farmers knew that ticks are the primary vector of this disease. It is interesting to highlight that 45.0% of the farmers that reported severe anaplasmosis did not use treatments against ticks. Climate change and the increase in global temperatures have favoured the survival of ticks in the environment, contributing to the spread of *A. ovis* as it occurs in other species, including humans. In an environment suitable for ticks, the rise in temperatures increases their survival and their period of activity, which, together with the coexistence of wild and domestic ruminants, provides a wide range of reservoirs and hosts, favouring the high prevalence of the disease [4,30]. 

Although the collective prevalence of *A. ovis* infection was 91.1%, only 40.4% of the studied farms showed severe anaplasmosis clinical signs, which affected mainly young animals. As has been reported in the literature, once the animals overcome the infection, if they have not been treated with antibiotics, they remain carriers for years, giving *A. ovis* PCR-positive results but showing no signs of disease [5,7]. Sick sheep showed severe anaemia, weakness, anorexia, cachexia and epiphora. Haematuria, being common in other haemoparasitosis, is not frequent in ovine anaplasmosis because it is a chronic process [2]. In our study, only 4.8% of the animals affected by severe anaplasmosis presented haematuria. In addition, the early culling of cachectic young animals was repeatedly reported by affected farmers (71.4%). Other authors also described this, highlighting the severe normocytic normochromic anaemia as the main symptom of this disease [5,7,31,32]. In agreement with these data, the present study found that 88.5% of the affected animals showed severe normochromic normocytic anaemia, with average values of erythrocytes of 6.7 (threshold values: 9–14 × 10^6^/mm^3^) and 22.7% of haematocrit (28–40%). It has been described that *Anaplasma* bacteria parasitise erythrocytes using a mechanism that is not well-defined but apparently non-lytic and that the destruction of erythrocytes and the consequent anaemia are a result of the immune response to anaplasma in the body [15].

While clinical signs of anaplasmosis were observed throughout the Matarraña region, severe clinical signs were seen much more frequently in farms located in the north of the province, with the risk of suffering severe anaplasmosis being 2.36-times higher in the north than in the south of the region (21/36 north vs. 0/15 south). Torina et al., 2008 [19] reported different *A. marginale, A. ovis* and *A. phagocytophilum* prevalences in the eastern and western sectors of the island of Sicily. These significant differences were related to differences in the management of the animals, to the diverse populations of wild reservoirs and especially to the variances between the most favourable habitats for the different species of ticks [19]. The Matarraña region has high mountain goat (*Capra pirenaica hispanica*) and roe deer populations (*Capreolus capreolus*), which could act as a reservoir for the disease. During the period of our study, 47 mountain goats were tested by means of qPCR in this area, and 19 of these (40.4%) tested positive for *A. ovis* (De la Fuente, personal communication). In addition, in the Matarraña region, there are two well-differentiated natural areas: the north, characterised by a continental climate with open spaces with small elevations, and the south, with a higher altitude and more humid climate [19]. Thus, differences were found in the presence of severe anaplasmosis according to the altitude at which the farms were located. Mountain goats and roe deer were initially only present in the more mountainous areas of the south. However, over the years, they have spread to the entire Matarraña region. Although *A. ovis* was present on practically all farms (91.1%), the northern farms were more severely affected. This could be because the disease entered silently from wild animals in the south; over time, it has spread throughout the region, with the greatest prevalence of clinical signs currently in the northern area due to the *A. ovis* infection-naïve population. Corona (2004) reported that anaplasmosis enzootic stability or endemic equilibrium occurs in warm climates where ticks are abundant throughout the year [33]. This implies the presence of a high percentage of infected animals, with the rare occurrence of clinical disease. As noted by the author, this relationship is maintained due to two factors: the passive immunity provided by colostrum and the early infection of the animals. This could explain the low rate of severe anaplasmosis in the South region of Matarraña. 

Finally, the influence of breed on the severity of anaplasmosis in sheep has also been reported in several studies [31,34,35]. However, in our study, no significant difference in the presence of severe anaplasmosis was found across breeds. 

## 5. Conclusions

Ovine anaplasmosis is an emerging disease in Europe that spreads rapidly through tick bites and is able of causing significant economic losses when it enters a naive area, causing an epidemic. However, there is a lack of epidemiological studies assessing the presence of *Anaplasma ovis* on small ruminant flocks. With the results obtained in the present study, it can be concluded that *Anaplasma ovis* rapidly spreads among all flocks in the affected area when management, climatic and environmental conditions are prone to the development of ticks, its primary vector. The data obtained in the present study also seem to indicate that as the disease becomes endemic in the area, the presence of clinical signs of the disease decreases. These results suggest that the detection of *A. ovis* by molecular techniques is insufficient to diagnose clinical anaplasmosis. Confirmation of the disease by clinical signs is needed, especially confirmation of a severe normocytic normochromic anaemia, the most diagnostic symptom of the disease.

## Figures and Tables

**Table 1:** Number of farms and animals sampled in each type of sheep farm analysed.

Farm Type	Number of Farms	Number of Samples
Type I	21	533
Type II	16	244
Type III	8	87
Total	45	864

**Table 2:** Percentage of farms with mild or severe anaplasmosis that reported have seen the following clinical signs in the animals—weakness, anorexia, cachexia, epiphora, haematuria, lameness and abortion—in comparison with those without anaplasmosis.

	Weakness	Anorexia	Cachexia	Epiphora	Haematuria	Lameness	Abortion
No anaplasmosis	4.3%	0%	4.3%	4.3%	0%	13%	8.7%
Mild anaplasmosis	100%	66.7%	83.3%	33.3%	0%	16.7%	16.7%
Severe anaplasmosis	100%	100%	100%	95.2%	4.8%	4.8%	23.8%

**Table 3:** Relationship between clinical signs of anaplasmosis and the breed reared on the farm.

Breed	Without Clinical Anaplasmosis	With Clinical Anaplasmosis
Rasa Aragonesa	12 (48.0%)	13 (52.0%)
Ojinegra de Teruel	4 (40.0%)	6 (60.0%)
Maellana	2 (33.0%)	4 (67.0%)

**Table 4:** Analysed farms with severe anaplasmosis clinical signs in the Matarraña region.

Farms	North Matarraña	South Matarraña
Severe anaplasmosis	21 (58.3%)	0 (0%)
Mild anaplasmosis	2 (5.6%)	4 (26.7%)
No anaplasmosis	13 (36.1%)	11 (73.3%)
Total	36	15

**Table 5:** Increase in the presence of ticks observed and absence of treatments against ticks in those farms that showed severe or mild anaplasmosis or absence of clinical signs.

	Increased Tick Presence	No Tick Treatment
No anaplasmosis	17.4%	27.3%
Mild anaplasmosis	50.0%	33.3%
Severe anaplasmosis	52.4%	45.0%

**Table 6:** Average values of the haematological analysis performed in sick animals. Measured parameters were leucocytes, erythrocytes, haemoglobin, haematocrit, platelets, mean corpuscular volume (MCV), mean corpuscular haemoglobin (MCH) and mean corpuscular haemoglobin concentration (MCHC).

Haematological Parameters	Average Values (n = 52)	Standard Error	Threshold Values
Leukocytes	5.9	0.37	4–12 × 10^3^/mm^3^
Erythrocytes	6.7	0.44	9–14 × 10^6^/mm^3^
Haematocrit	22.7	1.61	28–40%
Haemoglobin	7.4	0.50	8–15 g/dL
Platelets	451.2	40.28	250–750 × 10^3^/mm^3^
MCV	34.2	0.69	28–42 fL
MCH	10.8	0.13	8–12 pg
MCHC	31.7	0.32	30–34 g/dL

**Table 7:** *Anaplasma ovis* detection by PCR in the analysed farms in relation to the presence of clinical signs.

Farms	Pos. *Anaplasma ovis* PCR	Neg. *Anaplasma ovis* PCR
Clinical sings of anaplasmosis	21 (87.5%)	3 (12.5%)
No clinical sings	20 (95.2%)	1 (4.8%)
Total	41	4

## Data Availability

The data that support the findings of this study are available from the corresponding author upon request.
